# Dynamic Changes in Histone Modifications Are Associated with Differential Chromatin Interactions

**DOI:** 10.3390/genes15080988

**Published:** 2024-07-26

**Authors:** Yumin Nie, Mengjie Wang

**Affiliations:** Department of Bioinformatics, School of Biomedical Engineering and Informatics, Nanjing Medical University, Nanjing 211166, China

**Keywords:** differential chromatin interaction, histone modification, CTCF

## Abstract

Eukaryotic genomes are organized into chromatin domains through long-range chromatin interactions which are mediated by the binding of architectural proteins, such as CTCF and cohesin, and histone modifications. Based on the published Hi-C and ChIP-seq datasets in human monocyte-derived macrophages, we identified 206 and 127 differential chromatin interactions (DCIs) that were not located within transcription readthrough regions in influenza A virus- and interferon β-treated cells, respectively, and found that the binding positions of CTCF and RAD21 within more than half of the DCI sites did not change. However, five histone modifications, H3K4me3, H3K27ac, H3K36me3, H3K9me3, and H3K27me3, showed significantly more dramatic changes than CTCF and RAD21 within the DCI sites. For H3K4me3, H3K27ac, H3K36me3, and H3K27me3, significantly more dramatic changes were observed outside than within the DCI sites. We further applied a motif scanning approach to discover proteins that might correlate with changes in histone modifications and chromatin interactions and found that PRDM9, ZNF384, and STAT2 frequently bound to DNA sequences corresponding to 1 kb genomic intervals with gains or losses of a histone modification within the DCI sites. This study explores the dynamic regulation of chromatin interactions and extends the current knowledge of the relationship between histone modifications and chromatin interactions.

## 1. Introduction

Eukaryotic genomes are compacted into chromatin domains, including topologically associating domains (TADs) and A/B compartments, which play important roles in gene regulation during development and disease inside the nucleus. The two types of domains, TADs and compartments, reflect distinct mechanisms of the three-dimensional genome organization. TADs mediated by loop extrusion represent DNA regions within which physical interactions occur relatively frequently, while A/B compartments represent the spatial separation of transcriptionally active euchromatin and silent heterochromatin and distal interactions between genomic regions with the same type of chromatin modifications [1,2].

The formation of chromatin interactions and domains is mediated by the binding of specific proteins. As the main architectural protein of genome organization, CCCTC-binding factor (CTCF) restrains cohesin diffusion and loop extrusion to establish TAD boundaries [3] and is also able to organize long-range chromatin interactions between A compartments via RYBP-mediated phase separation [4]. Zinc finger protein 143 (ZNF143) [5] and myc proto-oncogene protein [6] affect the binding affinity of CTCF to DNA to mediate CTCF-bound chromatin loops. The ubiquitously expressed transcription factor Yin Yang 1 (YY1) binds to active enhancers and promoters and forms dimers to generate enhancer–promoter loops in a manner similar to CTCF [7]. Further, transcribing RNA polymerase II displaces CTCF and cohesin to disrupt chromatin interactions and remodels the genome organization downstream of response genes after influenza A virus (IAV) infection of human monocyte-derived macrophages (MDMs) [8].

CTCF, cohesin, and other architectural proteins contribute to the formation of chromatin interactions. However, acute CTCF, cohesin, and YY1 depletion minimally affected enhancer–promoter interactions in mouse embryonic stem cells, suggesting that histone modifications, chromatin remodeling, and other epigenetic factors might play crucial roles in the maintenance of enhancer–promoter interactions and higher-order chromatin structure [9]. Indeed, mounting evidence indicates direct links between histone modifications and higher-order chromatin structure. H3K4me1, H3K4me3, and H3K27ac have been shown to bookmark the TAD boundaries across mitosis [10], while elimination of H4K20me1 reduces X chromosome compaction and disrupts X chromosome conformation by weakening TAD boundaries [11]. Further, H3K9me2 and H3K9me3 regulate chromatin compartmentalization to form heterochromatin via phase separation [12] and the condensin-driven formation of TADs on X chromosomes [13].

The formation and maintenance of chromatin interactions and domains are associated with the binding of specific proteins and histone modifications. A previous study indicated that IAV infection of MDMs causes readthrough transcription past the ends of response genes and the disruption of chromatin interactions by the removal of cohesin at CTCF sites downstream of genes [8]. However, it is still not known how the binding of CTCF and cohesin and histone modifications are associated with changes in chromatin interactions that are not caused by readthrough transcription. Here, based on the published Hi-C and ChIP-seq datasets in mock-, IAV-, and interferon β (IFN-β)-treated human MDMs, we identified the significant differential chromatin interactions (DCIs) and investigated in detail how the binding of CTCF and cohesin and histone modifications change within DCI sites after removing those within transcription readthrough regions. We found that gains or losses of CTCF and RAD21 (a component of the cohesin complex) binding only occurred within less than 40% of DCI sites, and dramatic changes in histone modifications occurred within and outside DCI sites. We further divided pairs of the DCI sites into 1 kb genomic intervals and found that histone-lysine N-methyltransferase PRDM9, zinc finger protein 384 (ZNF384), and signal transducer and activator of transcription 2 (STAT2) frequently bound within intervals with gains or losses of a histone modification within the DCI sites.

## 2. Materials and Methods

### 2.1. Data Source

Raw paired-end reads for six in situ Hi-C experiments in mock-, IAV-, and IFN-β-treated human MDMs at 6 h after infection were downloaded from the NCBI Gene Expression Omnibus under the accession number GSE103477 [8]. Raw reads for each of two replicates per experiment condition were first trimmed from the 3′ end of sequences to GATC and mapped to the human reference genome hg19 using Bowtie2 software (v 2.5.0) [14]. Aligned reads with a mapping quality lower than 10 and multi-mapped reads were then removed, and read pairs with the exact same ends were only considered once to eliminate PCR duplicates. The HOMER (http://homer.ucsd.edu/homer) (accessed on 24 May 2023) program makeTagDirectory was finally used twice to generate tag directories from aligned reads, and a second run of makeTagDirectory would remove small fragments and self-ligations with the parameters-removePEbg-removeSpikes 10000 5.

ChIP-seq reads and peaks for CTCF, RAD21, and five histone modifications (H3K4me3, H3K27ac, H3K36me3, H3K9me3, and H3K27me3) in mock-, IAV-, and IFN-β-treated human MDMs at 6 h after infection were also downloaded from the NCBI Gene Expression Omnibus under the accession number GSE103477 [8]. For each of the histone modifications, raw ChIP-seq reads were first mapped to the hg19 genome using Bowtie2 software [14], and every uniquely mapped read was then shifted by 100 bp in the 5′ to 3′ direction. The start positions of the reads were finally used to compute the levels of histone modifications within 1 kb genomic intervals.

### 2.2. Identification of DCIs

Hi-C contact matrices in hic format were generated from HOMER tag directories using the HOMER program tagDir2hicFile and further converted to the sparse upper triangular format using the straw program with the normalization method of square root of vanilla coverage at 50 kb resolution [15]. Fast loess joint normalization was then implemented between two experimental conditions, and differences between Hi-C datasets were detected using the quasi-likelihood F-test method within the multiHiCcompare package [16]. DCIs were finally considered as pairs of 50 kb bins with adjusted *p*-values of less than 0.05, fold changes in normalized contact counts greater than 2, and genomic distances greater than 150 kb. Hi-C contact maps and DCIs were visualized using Juicebox software (v1.11.08) [15]. According to the definition of the resolution of a Hi-C experiment as the smallest bin size at which 80% of bins have at least 1000 contacts [17], the resolutions of the Hi-C experiments in mock-, IAV-, and IFN-β-treated human MDMs were 50 kb, 30 kb, and 35 kb, respectively. The human genome was thus divided into non-overlapping 50 kb bins, and DCIs were identified at the 50 kb resolution.

### 2.3. Binarization of ChIP-Seq Data for Transcription Factors and Histone Modifications

A pair of 50 kb genomic regions where a DCI occurred were divided into 1 kb non-overlapping intervals. A transcription factor, whose peaks overlapped with a 1 kb interval within the DCI sites, was considered to bind within the interval. For each of the histone modifications, the start positions of reads that overlapped with a 1 kb interval were counted using a window size of 60 bp, and a histone modification with more reads than the threshold value was considered to be present within the interval. The threshold, *t*, for each of the histone modifications was based on the total number of mapped reads and was set to be the smallest integer *t* such that P(*X* > *t*) < 10^−4^, where *X* is a random variable, with a Poisson distribution with the mean parameter set to the expected number of reads mapped to the 60 bp window by random chance [18], which was calculated as follows:

(Number of mapped reads) × 60/(genome size × 0.9)

For each of the transcription factors and histone modifications, a 100-dimensional binary vector was constructed for each of DCIs, and the interval within which a transcription factor or histone modification was present was set to 1. The binarized ChIP-seq data were considered as symmetric binary variables, and the proportions of intervals having different values were applied to measure the changes in transcription factor binding and histone modifications after IAV infection and IFN-β treatment.

### 2.4. Statistical Significances of Changes in Transcription Factor Binding and Histone Modifications

The Wilcoxon rank-sum test was used to determine whether there were significant changes in transcription factor binding and histone modifications after IAV infection and IFN-β treatment. The default significance level was set to 0.05 unless stated otherwise in our results.

### 2.5. Binding of Transcription Factors within DCI Sites

For each pair of DCI sites, DNA sequences corresponding to the 1 kb intervals with gains or losses of a histone modification were first extracted and scanned using FIMO software (version 5.5.4) with a *p*-value threshold of 0.0001 [19]. Position weight matrices of binding motifs for 841 vertebrate transcription factors were downloaded from the JASPAR CORE database [20]. The occurrence number of each transcription factor was then counted, and transcription factors that most frequently bound within intervals were visualized using Cytoscape software (version 3.7.2) [21].

## 3. Results

### 3.1. Identification of DCIs

**Figure 1:**
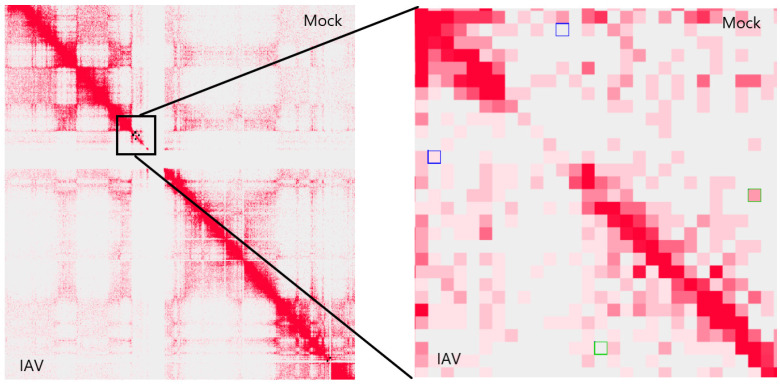
Hi-C contact maps of mock- and IAV-treated MDMs and two DCIs on chromosome 11. The blue and green square represented a strengthened and weakened DCI, respectively.

Compared with mock infection, 452 chromatin interactions were differentially regulated after IAV treatment of MDMs (*p* < 0.05, fold change > 2, interaction distance > 150 kb), among which 136 were strengthened and 316 were weakened. There were still 102 strengthened and 104 weakened DCIs after removing those within transcription readthrough regions, suggesting that other factors except for transcription elongation affect the chromatin structure. In order to eliminate the effect of transcription elongation on chromatin structure, our subsequent analyses were based on the 206 DCIs that were not located within IAV-induced readthrough regions. Further, IFN-β stimulation of MDMs did not affect the chromatin structure downstream of genes, and we identified 55 strengthened and 72 weakened DCIs after IFN-β treatment of MDMs. As an example, a strengthened and a weakened DCI on chromosome 11 in an IAV-infected MDM are shown below (Figure 1).

**Figure 2:**
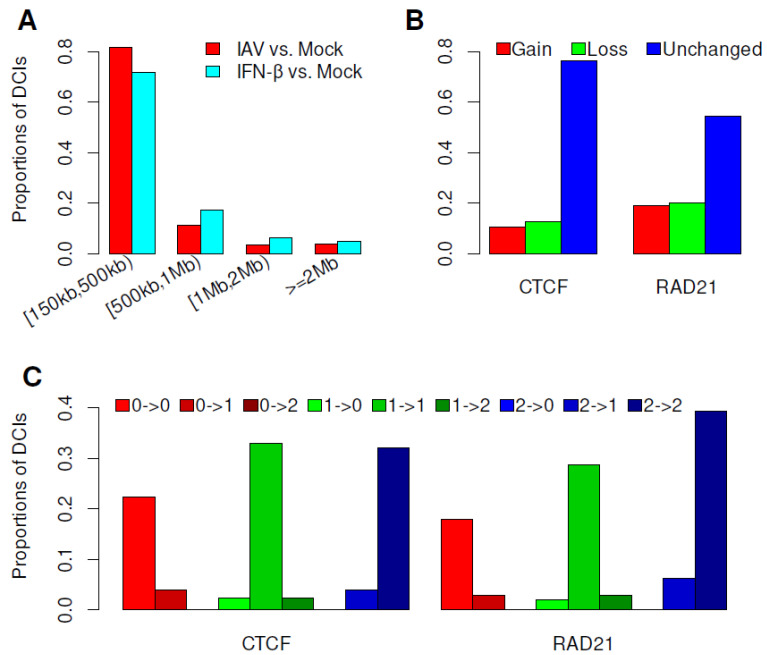
Genomic distances between DCI sites and patterns of CTCF and RAD21 binding within DCI sites after IAV infection. (**A**) Proportions of DCI sites with different distances after IAV and IFN-β treatment of MDMs. (**B**) Gains and losses of CTCF and RAD21 binding within DCI sites after IAV infection. (**C**) Changes in the patterns of CTCF and RAD21 binding within DCI sites after IAV infection. For two genomic regions where a DCI occurred, 0, 1, and 2 represent binding peaks of CTCF or RAD21 overlapped with neither, either, and both of the genomic regions, respectively.

We calculated the distances between two genomic regions where DCIs occurred and found that a large proportion of DCIs (93% after IAV infection and 89% after IFN-β treatment) were local interactions along the diagonal of the Hi-C contact map, whose distances ranged in size from 150 kb to 1 Mb (Figure 2A). The binding of proteins and their interactions within TADs and compartments might contribute to these changes in chromatin interactions after IAV infection and IFN-β treatment of MDMs [2].

### 3.2. Binding of CTCF and RAD21 within DCI Sites

CTCF and cohesin organize the genome into chromatin loops, and loss of CTCF or cohesin would eliminate chromatin loops and affect the local genome structure [1,22]. Our analyses indicated that less than 40% of the DCIs showed gains or losses of CTCF and RAD21 binding, and the binding positions of CTCF and RAD21 within 76.2% and 54.4% of DCIs, respectively, did not change after IAV infection (Figure 2B). Specifically, only 13.5% and 21.6% of weakened loops after IAV infection showed losses of CTCF and RAD21 binding, respectively, while 14.7% and 18.6% of strengthened loops showed gains of CTCF and RAD21 binding, respectively. We further investigated the patterns of CTCF and RAD21 binding at two genomic regions where a DCI occurred and found that 22.3% and 18.0% of IAV-induced DCIs contained no CTCF and RAD21 binding peaks, respectively, in both mock- and IAV-infected MDMs (Figure 2C).

The analyses of IFN-β treatment of MDMs led to the same conclusion. More than 70% of weakened and strengthened loops showed no changes in CTCF and RAD21 binding, among which more than 51% of DCI sites contained no binding peaks of CTCF and RAD21 (Figure S1). All these results suggest that the changes in chromatin interactions after IAV infection and IFN-β treatment of MDMs were partially dependent on the gains or losses of CTCF and RAD21 binding, and other factors including compartmentalization might play important roles in mediating chromatin structure.

### 3.3. Dramatic Changes in Histone Modifications within and outside DCI Sites

**Figure 3:**
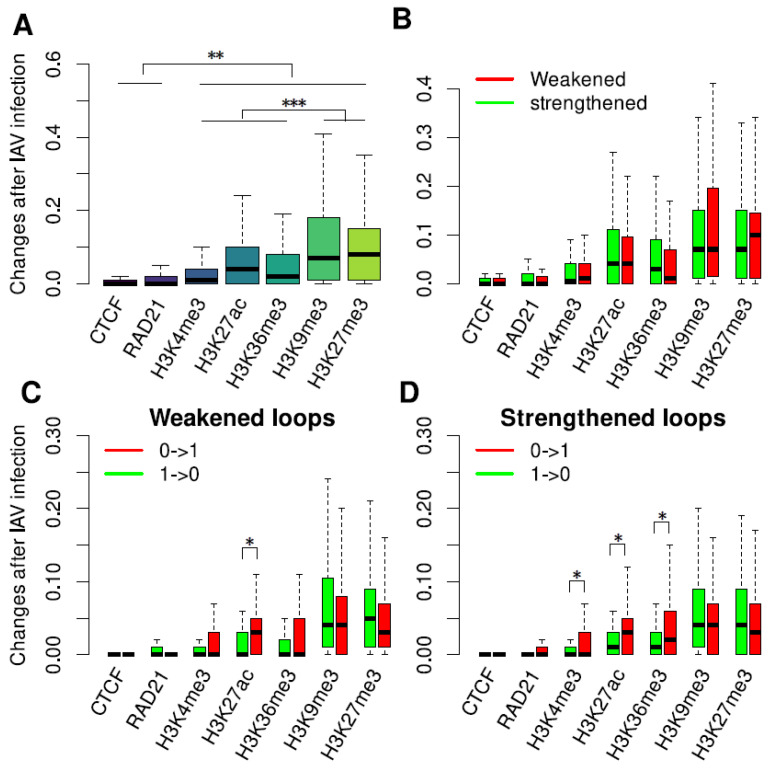
Changes in the binding of CTCF and RAD21 and histone modifications within DCI sites after IAV infection. (**A**) Changes within all DCI sites. (**B**) Changes within weakened and strengthened loops. (**C**,**D**) Gains and losses of CTCF and RAD21 binding and histone modifications within weakened and strengthened loops. Here, 0 and 1 represent a transcription factor or histone modification being absent or present within an interval, respectively. *, **, and *** represent a *p*-value of less than 0.05, 0.005, and 0.001, respectively.

In order to investigate in detail how the binding of CTCF and RAD21 and histone modifications changed within the DCI sites, we divided pairs of 50 kb genomic regions where DCIs occurred into 1 kb non-overlapping intervals and then determined whether CTCF, RAD21, or a histone modification were present within each of intervals. Compared with mock infection, changes in the binding of CTCF and RAD21 and histone modifications after IAV infection and IFN-β treatment were finally measured by the proportions of intervals having different binary values. Our results indicated that all five histone modifications, H3K4me3, H3K27ac, H3K36me3, H3K9me3, and H3K27me3, showed significantly more dramatic changes than CTCF and RAD21 (Wilcoxon rank-sum test, *p* < 0.005), and the two repressive histone modifications, H3K9me3 and H3K27me3, showed significantly more dramatic changes than the three active histone modifications, H3K4me3, H3K27ac, and H3K36me3 (Wilcoxon rank-sum test, *p* < 0.001), within the DCI sites after both IAV and IFN-β treatment of MDMs (Figure 3A and Figure S2A). We further classified the DCIs into weakened and strengthened loops and found that changes in the binding of CTCF and RAD21 and histone modifications showed no significant differences between the weakened and strengthened loops after both IAV and IFN-β treatment of MDMs (Figure 3B and Figure S2B). However, weakened loops contained significantly more intervals where H3K27ac became present, and strengthened loops contained significantly more intervals where H3K4me3, H3K27ac, and H3K36me3 became present after IAV infection (Figure 3C,D). Both weakened and strengthened loops contained significantly more intervals where H3K9me3 became absent after IFN-β treatment (Figure S2C,D).

**Figure 4:**
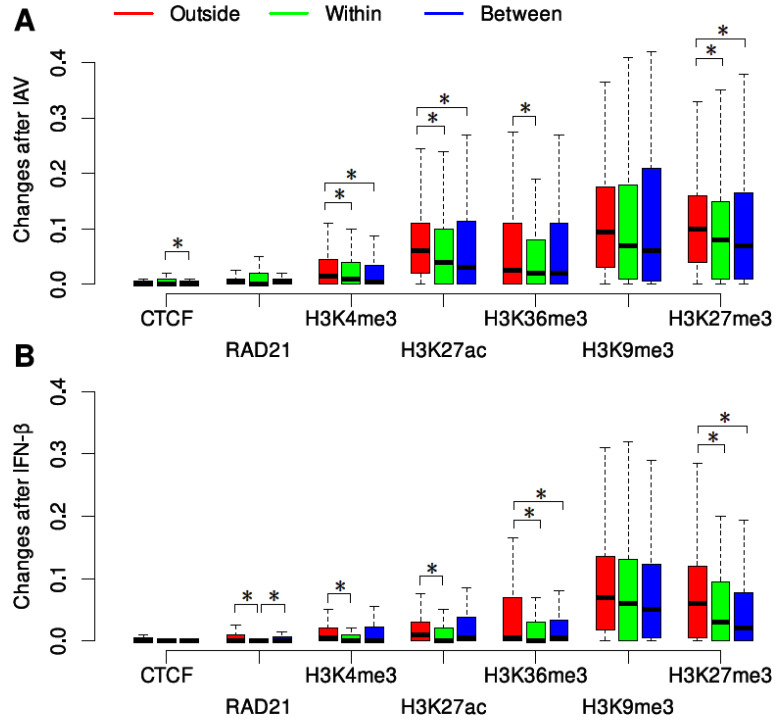
Changes in the binding of CTCF and RAD21 and histone modifications around DCI sites after IAV infection (**A**) and IFN-β treatment (**B**). * *p*-value of less than 0.05 produced by a Wilcoxon rank-sum test.

In order to further investigate how the binding of CTCF and RAD21 and histone modifications changed surrounding the DCI sites, 200 kb genomic regions, corresponding to regions of four bins, outside and between the DCI sites were also divided into 1 kb non-overlapping intervals to compute the proportions of intervals with gains or losses of transcription factor binding and histone modifications. If the distance between a pair of DCI sites was less than 200 kb, the whole region between the DCI sites was considered. Compared to changes within DCI sites, CTCF binding showed significantly fewer changes between DCI sites after IAV infection (Figure 4A), and RAD21 binding showed significantly more changes outside and between DCI sites after IFN-β treatment (Figure 4B), suggesting that CTCF and RAD21 play distinct roles in affecting chromatin interactions in different cell conditions. Considering the minimal changes in CTCF and RAD21 binding within and outside the DCIs, the significant changes in CTCF and RAD21 binding surrounding the DCIs might not be biologically meaningful. For each of the five histone modifications, changes within and between the DCI sites showed no significant differences in two conditions. However, significantly more dramatic changes were observed outside the DCI sites for H3K4me3, H3K27ac, H3K36me3, and H3K27me3 after both IAV and IFN-β treatment (Figure 4). These results suggest that dramatic changes in histone modifications occurred not only within the DCI sites but also outside the pairs of DCI sites.

### 3.4. Binding of Transcription Factors within DCI Sites

Dramatic changes in histone modifications within DCI sites might result from the binding of specific proteins. In order to discover new proteins that might correlate with the changes in histone modifications and chromatin interactions, DNA sequences corresponding to 1 kb genomic intervals with gains or losses of a histone modification within DCI sites were extracted and scanned using position weight matrices of transcription factor binding motifs to determine whether a transcription factor was bound within an interval. For each of the histone modifications, intervals containing binding motifs of a specific transcription factor were counted, and the top five transcription factors that most frequently bound within intervals were obtained. We found that for all five histone modifications, PRDM9, ZNF384, and STAT2 frequently bound within intervals after both IAV and IFN-β treatment, suggesting that these three transcription factors might mediate changes in histone modifications and chromatin interactions in a universal way. PRDM9 and ZNF384 are both C2H2-type zinc finger proteins, and both STAT protein and zinc-coordinating transcription factors are capable of dimerization [23]. Dimerization of these transcription factors or their interactions with other proteins might contribute to the dynamic changes in histone modifications and chromatin interactions. Further, PRDM9 is a histone methyltransferase that trimethylates H3K4 and H3K36 [24], and the binding of PRDM9 can directly mediate levels of H3K4me3 and H3K36me3 within intervals.

**Figure 5:**
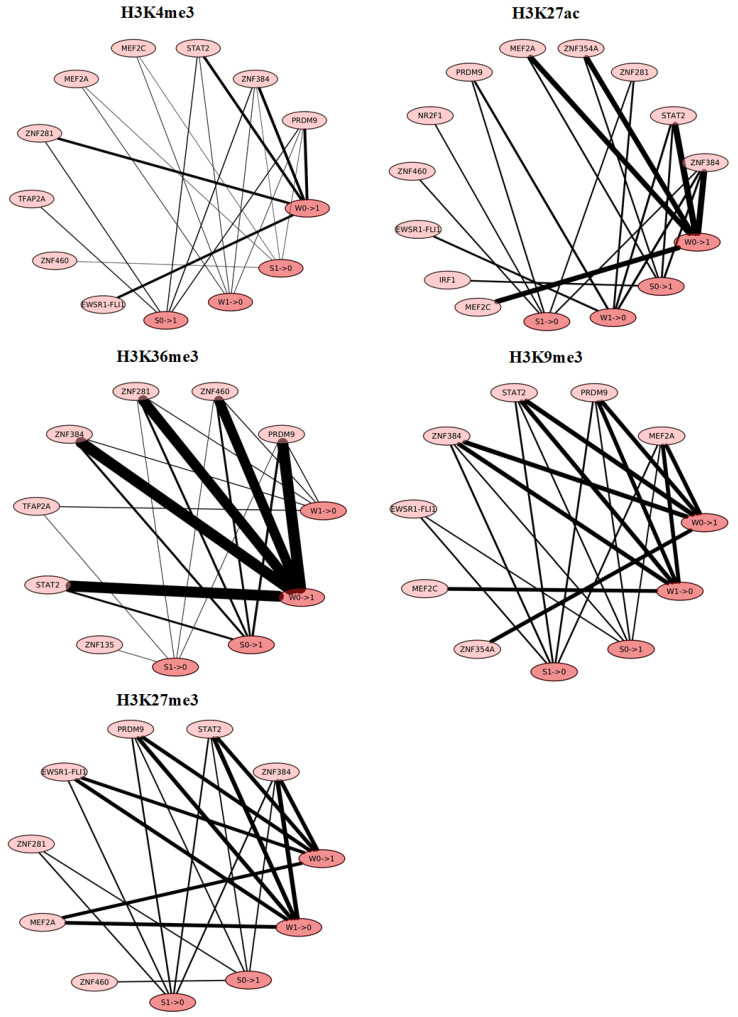
Transcription factors most frequently bound within four types of intervals within DCI sites after IAV infection. Nodes represent transcription factors and four types of intervals. For each type of interval, W and S represent weakened and strengthened loops, respectively, while 0→1 and 1→0 represent gain and loss of a histone modification, respectively. The width of the lines between transcription factors and types of intervals is proportional to the occurrence number of transcription factors within the intervals.

Based on the changes in histone modifications (gain or loss) and chromatin interactions (weakened or strengthened), genomic intervals with gains or losses of a histone modification were further classified into four types, and the top five transcription factors that most frequently bound within each type of interval were visualized for each of the histone modifications. Our analyses indicated that the transcription factors that frequently bound within each type of interval differed widely, especially for the two active histone modifications H3K4me3 and H3K27ac (Figure 5 and Figure S3), suggesting that the gains and losses of a histone modification within weakened or strengthened loops were associated with different transcription factors. However, several transcription factors were found to frequently bind within all four types of intervals. For example, PRDM9, ZNF384, zinc finger protein 460 (ZNF460), myocyte-specific enhancer 2A (MEF2A), and STAT2 frequently bound within intervals where H3K4me3, H3K27ac, H3K36me3, H3K9me3, and H3K27me3 changed, respectively, in both strengthened and weakened loops after IAV infection (Figure 5), while interferon regulatory factor 1 (IRF1), PRDM9, STAT2, and ZNF384 frequently bound within intervals where H3K27ac, H3K36me3, H3K9me3, and H3K27me3 changed, respectively, in both strengthened and weakened loops after IFN-β treatment (Figure S3).

### 3.5. Colocalization of CTCF, RAD21, and Histone Modifications

**Figure 6:**
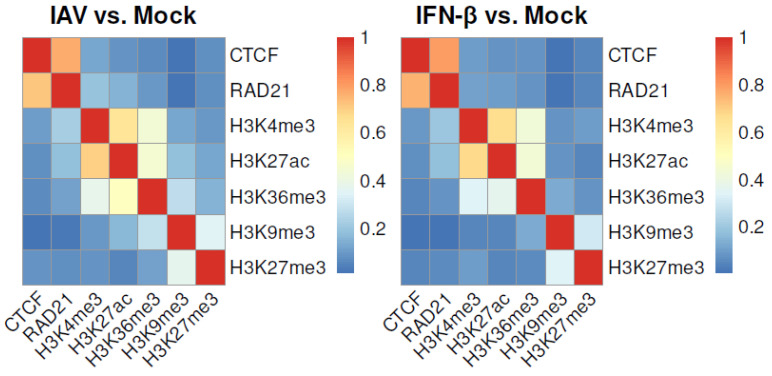
Colocalization of CTCF, RAD21, and five histone modifications in mock-, IAV-, and IFN-β-treated MDMs. The lower and upper triangular heat maps show pairwise similarities in mock and IAV (or IFN-β) conditions, respectively.

Transcription factors cooperatively bind to DNA, and histone modifications coexist to regulate gene transcription [25]. In order to measure correlations between the locations of transcription factors and histone modifications, the cosine similarity function was applied to calculate pairwise similarities of CTCF, RAD21, and the five histone modifications based on the binarized ChIP-seq data in mock-, IAV-, and IFN-β-treated MDMs. A higher cosine similarity indicated a higher degree of colocalization between two transcription factors or histone modifications. We observed a strong correlation between the binding locations of CTCF and RAD21 and moderate pairwise correlations between three active histone modifications and between two repressive histone modifications in three conditions (Figure 6). Specifically, the similarity between H3K4me3 and H3K27ac was larger than 0.63 in three conditions. The higher similarities between active or repressive histone modifications might partially explain the overlaps between transcription factors that frequently bound within intervals with gains or losses of a histone modification.

## 4. Discussion

Multiple factors, including architectural proteins and histone modifications, contribute to the formation and maintenance of chromatin interactions and domains. CTCF and cohesin are architectural proteins that function together to form chromatin loops and TADs, while interactions between genomic regions with the same types of histone modifications contribute to the formation of A/B compartments [2]. We first investigated how the binding of CTCF and RAD21 changed within DCI sites and found that the binding positions of CTCF and RAD21 within more than half of the DCI sites did not change, suggesting that changes in chromatin interactions are mostly CTCF-independent and compartmentalization may play a prominent role in changes in chromatin interactions after IAV and IFN-β treatment.

The further analyses of histone modifications within DCI sites indicated that both active and repressive histone modifications showed more dramatic changes than CTCF and RAD21 within the DCI sites, and the two repressive histone modifications H3K9me3 and H3K27me3 showed significantly more dramatic changes than the three active histone modifications H3K4me3, H3K27ac, and H3K36me3. For H3K4me3, H3K27ac, H3K36me3, and H3K27me3, more dramatic changes were observed outside but not between a pair of DCI sites. These results suggest that changes in chromatin interactions after IAV and IFN-β treatment are highly associated with changes in histone modifications within and outside pairs of DCI sites. H3K9me3 and H3K27me3 are repressive histone modifications that have been confirmed to promote the formation of genomic compartments [13,26]. H3K4me3, H3K27ac, and H3K36me3 are active histone modifications that are enriched in promoter regions, active enhancers, and actively transcribed genes, respectively, and changes in these histone modifications may affect interactions between enhancers and promoters and the formation of genomic compartments [27]. Previous studies have shown that interactions between heterochromatic regions rather than euchromatic regions are energetically favorable and crucial for establishing compartmentalization [28]. The significantly more dramatic changes in the heterochromatin-associated histone modifications H3K9me3 and H3K27me3 thus suggest that the two repressive histone modifications may play important roles in modulating genome compartmentalization and shaping the genome organization in IAV- and IFN-β-treated MDMs. Further, both repressive and active histone modifications have been demonstrated to correlate with the formation of TADs, which might also contribute to changes in chromatin interactions in IAV- and IFN-β-treated MDMs [10,13].

In addition to CTCF and cohesin, several other proteins, including YY1 [7] and ZNF143 [5], also mediate chromatin interactions in mammals. We found that PRDM9, ZNF384, and STAT2 frequently bound to DNA sequences corresponding to 1 kb genomic intervals with gains or losses of a histone modification within the DCI sites, suggesting that these transcription factors might be associated with changes in histone modifications and chromatin interactions. The three transcription factors are all capable of dimerization, which might mediate the formation of chromatin interactions. We further found that gains and losses of a histone modification within the DCI sites were associated with different transcription factors, suggesting that specific transcription factors cooperate to affect histone modifications and chromatin interactions within different chromatin contexts. However, it still remains unknown how these transcription factors affect histone modifications and/or chromatin interactions. As a motif scanning approach was used to discover proteins that frequently bound within DCI sites, cofactors, including mediators that lack DNA binding capabilities, were not considered in our analyses. It has been suggested that mediators also participate in the formation of long-range chromatin interactions and heterochromatin [29,30]. ChIP-seq data for cofactors, which were not available in mock-, IAV-, and IFN-β-treated MDMs, would help to investigate the relationship between cofactors and changes in chromatin interactions.

## 5. Conclusions

In this study, we identified DCIs in IAV- and IFN-β-treated human MDMs and investigated how the binding of CTCF and RAD21 and histone modifications changed within DCI sites after removing those within transcription readthrough regions. We found that gains or losses of CTCF and RAD21 binding only occurred within less than 40% of DCI sites, and the five histone modifications H3K4me3, H3K27ac, H3K36me3, H3K9me3 and H3K27me3 showed significantly more dramatic changes than CTCF and RAD21 within the DCI sites. Compared with changes within the DCI sites, H3K4me3, H3K27ac, H3K36me3, and H3K27me3 showed significantly more dramatic changes outside but not between pairs of DCI sites. We further found that PRDM9, ZNF384, and STAT2 frequently bound to DNA sequences corresponding to 1 kb genomic intervals with gains or losses of a histone modification within DCI sites. These results provide an insight into the relationship between histone modifications and chromatin interactions and demonstrate that dynamic changes in histone modifications are highly associated with changes in chromatin interactions, which suggests that histone modifications might play an important role in the formation and maintenance of chromatin interactions and domains.

## Data Availability

All data analyzed during the current study are included in this published article.

## Supplementary Materials

The following supporting information can be downloaded at: https://www.mdpi.com/article/10.3390/genes15080988/s1, Figure S1: Patterns of CTCF and RAD21 binding within DCI sites after IFN-β treatment; Figure S2: Changes in the binding of CTCF and RAD21 and histone modifications within DCI sites after IFN-β treatment; Figure S3: Transcription factors most frequently bound within four types of intervals within DCI sites after IFN-β treatment.
