# Supplementary figures and images for: Dynamic Changes in Histone Modifications Are Associated with Differential Chromatin Interactions
Source: Genes (Basel). 2024 Jul 26;15(8):988. doi: 10.3390/genes15080988 (PMC11353334; doi:10.3390/genes15080988)

**A** Weakened loops after IFN- $\beta$ 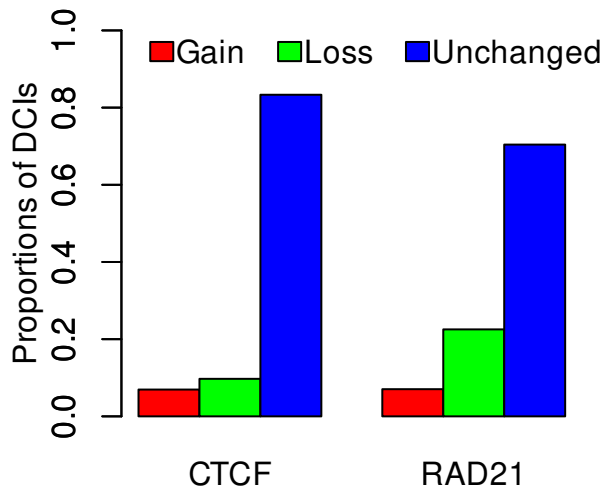**B** Strengthened loops after IFN- $\beta$ 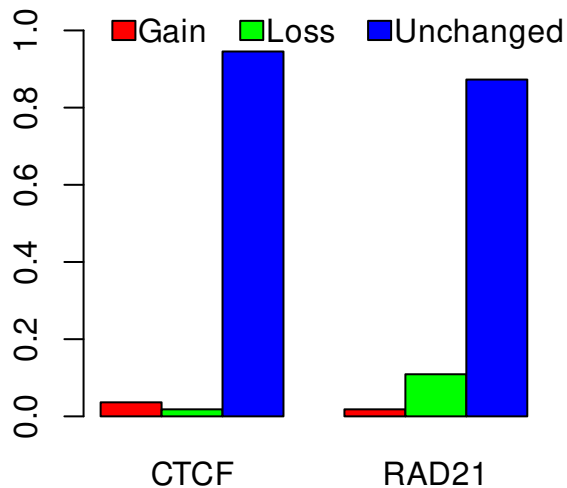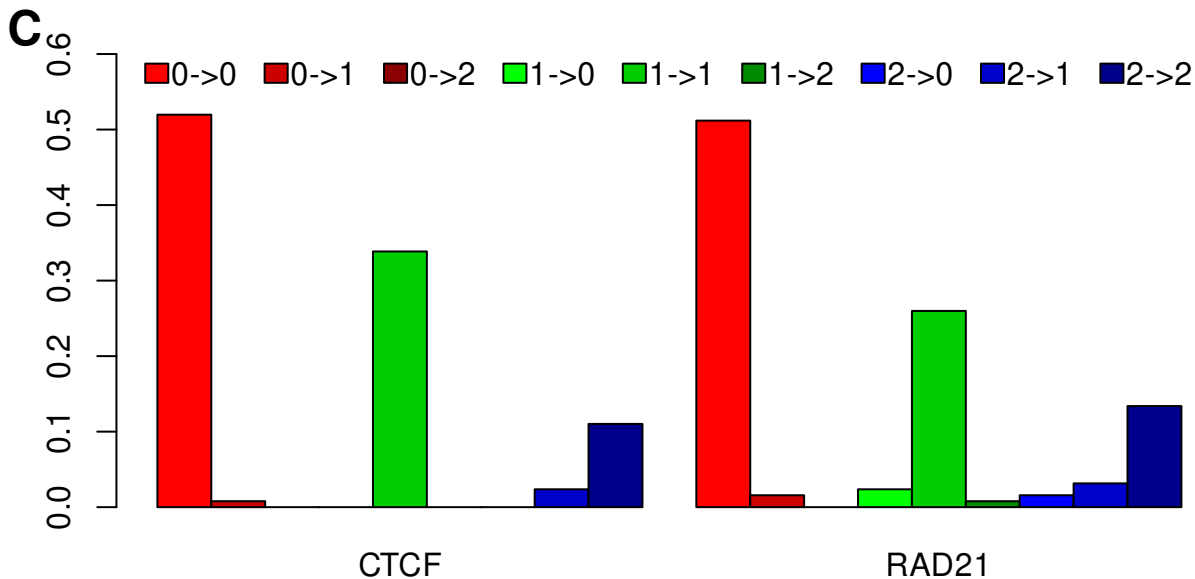

Supplement: Supplementary file 1 [file genes-15-00988-s001.zip › Figure S1.pdf]

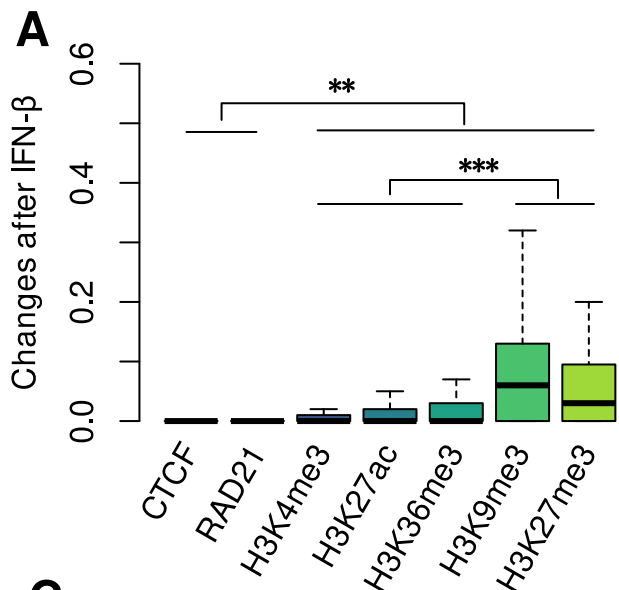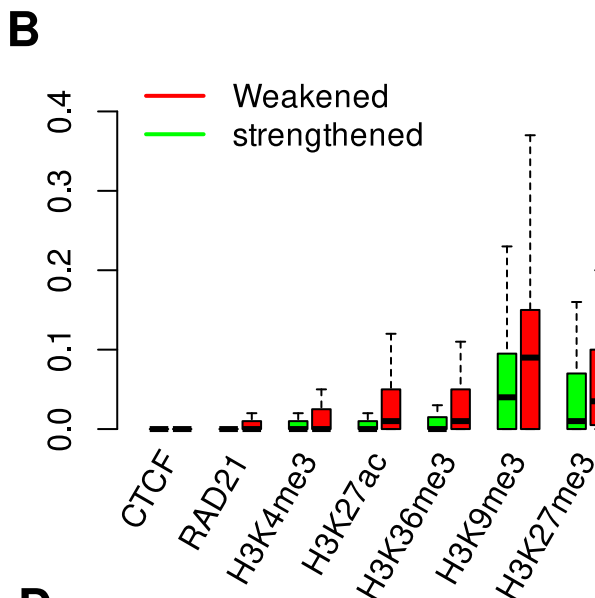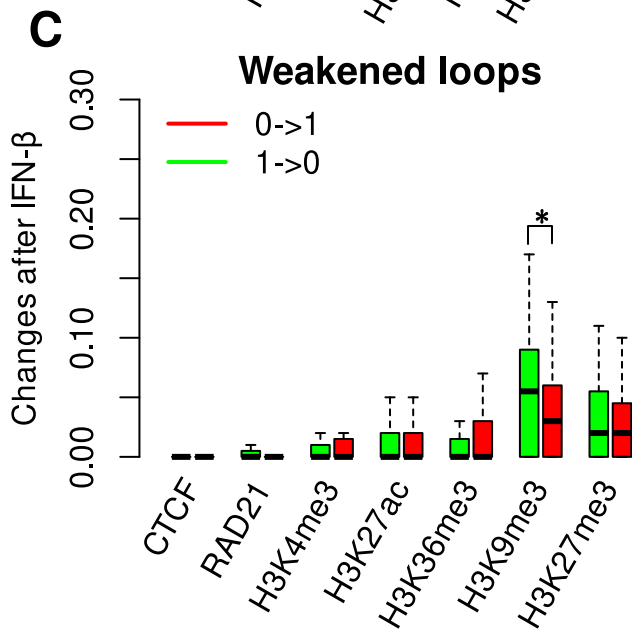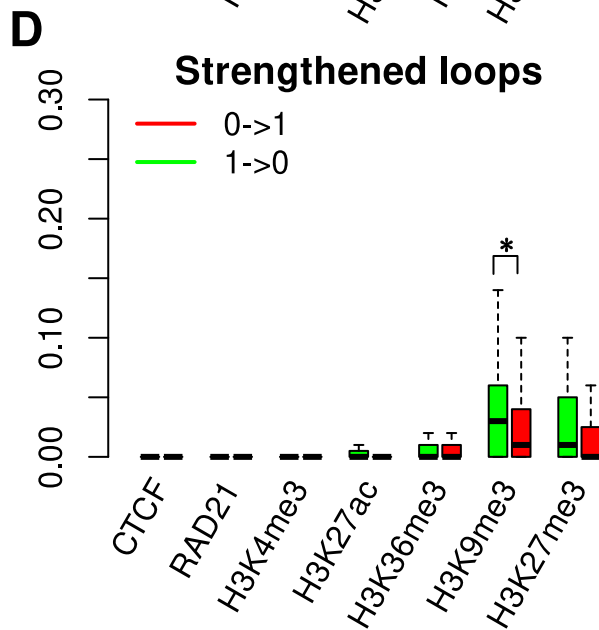

Supplement: Supplementary file 1 [file genes-15-00988-s001.zip › Figure S2.pdf]

### H3K4me3

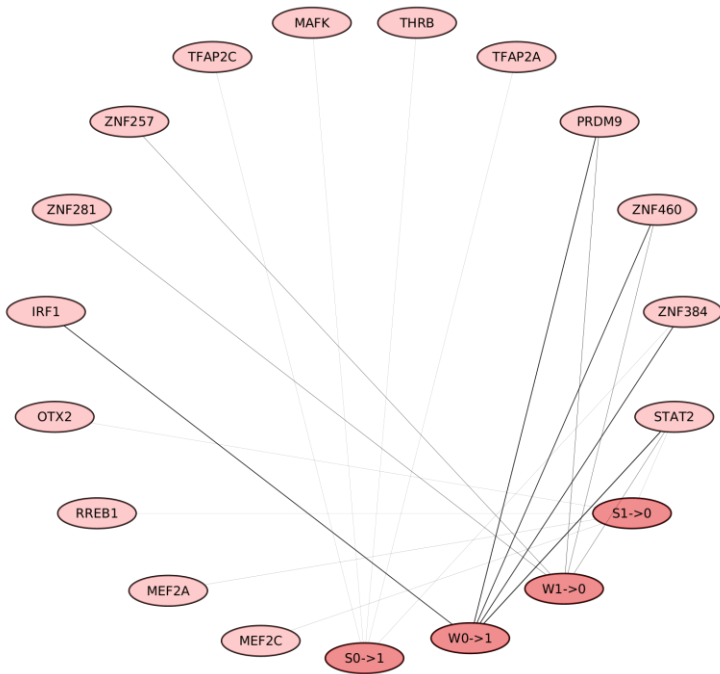

### H3K36me3

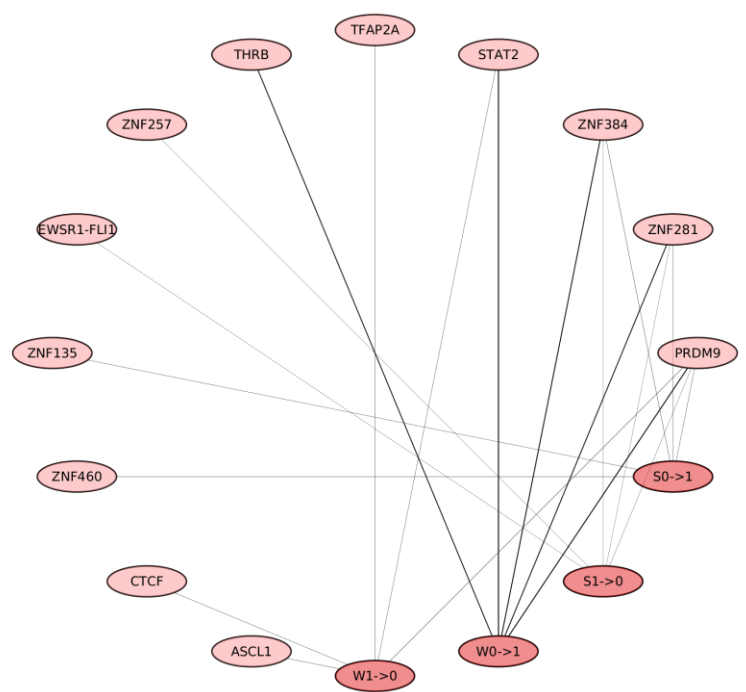

### H3K27ac

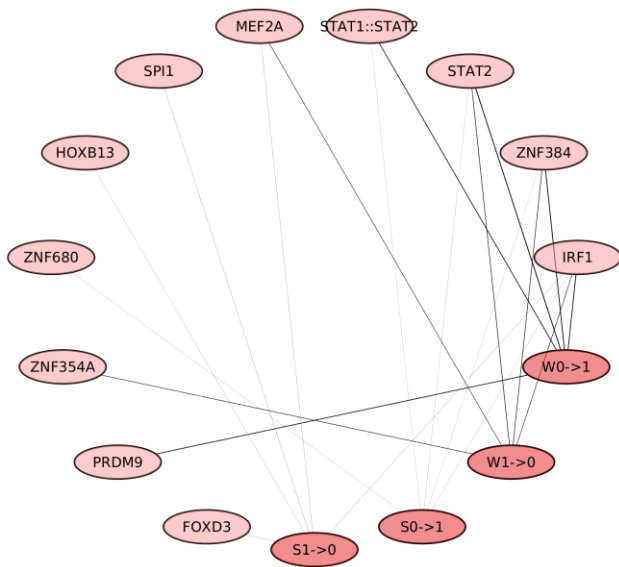

### H3K9me3

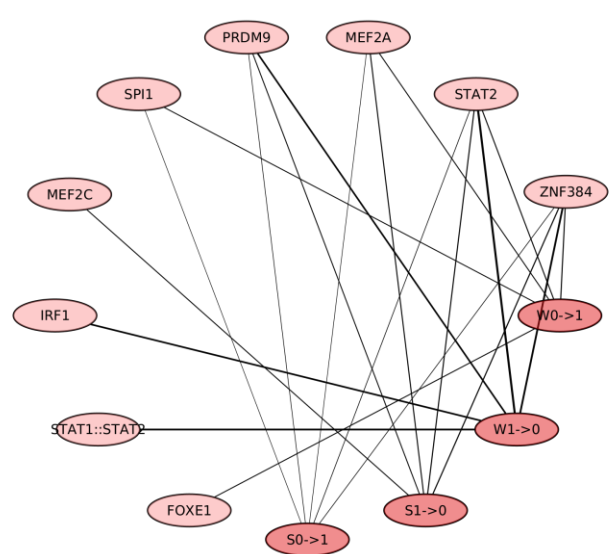

### H3K27me3

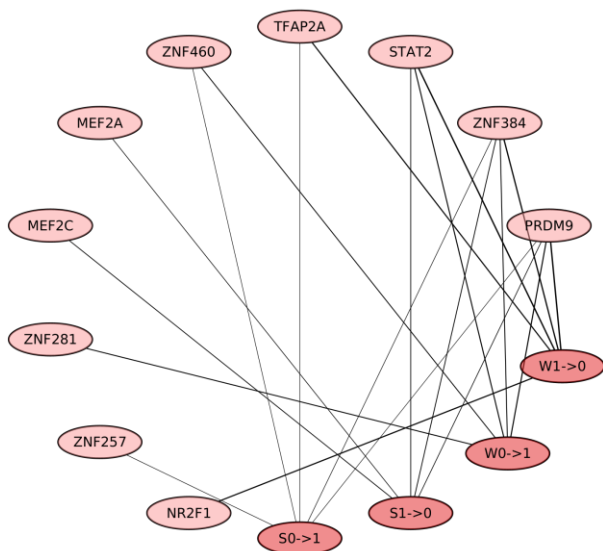

Supplement: Supplementary file 1 [file genes-15-00988-s001.zip › Figure S3.pdf]
